# Automatic tongue image quality assessment using a multi-task deep learning model

**DOI:** 10.3389/fphys.2022.966214

**Published:** 2022-09-20

**Authors:** Huimin Xian, Yanyan Xie, Zizhu Yang, Linzi Zhang, Shangxuan Li, Hongcai Shang, Wu Zhou, Honglai Zhang

**Affiliations:** ^1^ School of Medical Information Engineering, Guangzhou University of Chinese Medicine, Guangzhou, China; ^2^ Ministry of Education and Beijing Key Laboratory of Internal Medicine of Traditional Chinese Medicine, Beijing University of Chinese Medicine, Beijing, China

**Keywords:** multi-task learning model, tongue image quality assessment, tongue segmentation, traditional Chinese medicine, deep learning

## Abstract

The quality of tongue images has a significant influence on the performance of tongue diagnosis in Chinese medicine. During the acquisition process, the quality of the tongue image is easily affected by factors such as the illumination, camera parameters, and tongue extension of the subject. To ensure that the quality of the collected images meet the diagnostic criteria of traditional Chinese Medicine practitioners, we propose a deep learning model to evaluate the quality of tongue images. First, we acquired the tongue images of the patients under different lighting conditions, exposures, and tongue extension conditions using the inspection instrument, and experienced Chinese physicians manually screened them into high-quality and unqualified tongue datasets. We then designed a multi-task deep learning network to classify and evaluate the quality of tongue images by adding tongue segmentation as an auxiliary task, as the two tasks are related and can promote each other. Finally, we adaptively designed different task weight coefficients of a multi-task network to obtain better tongue image quality assessment (IQA) performance, as the two tasks have relatively different contributions in the loss weighting scheme. Experimental results show that the proposed method is superior to the traditional deep learning tongue IQA method, and as an additional task of the network, it can output the tongue segmentation area, which provides convenience for follow-up clinical tongue diagnosis. In addition, we used network visualization to verify the effectiveness of the proposed method qualitatively.

## 1 Introduction

Tongue diagnosis is one of the most important diagnostic methods in traditional Chinese medicine, and it provides an effective, non-invasive criteria to assist in the assessment of a patient’s physical condition (Li et al., 2019; Xie et al., 2021). Traditional tongue diagnosis is affected by objective and subjective factors, such as the external light environment and the clinical experience of practitioners. With the development of computer information technology, through computer imaging of the tongue in a stable environment, tongue images can be digitally and quantitatively studied based on image processing technology, thus, making the process of tongue diagnosis more objective and standardized. However, the tongue imaging process is inevitably affected by factors such as changes in illumination, camera parameters, and the protruding posture of the tongue, which greatly influence the quality of the tongue image, thereby affecting the performance of subsequent tongue diagnosis. Therefore, evaluating the quality of obtained tongue images has become an important and indispensable part of tongue diagnosis.

Image quality assessment (IQA) is a method to evaluate objective image quality consistent with human subjective judgments (Liu et al., 2019). At present, the clinical evaluation of tongue image quality mainly relies on the doctor’s senses and clinical experiences; for example, the illumination is uniform, the color is not distorted, there is no artifact, the tongue is fully stretched, etc. Therefore, it can be concluded that the traditional evaluation process of clinical tongue image quality has the following shortcomings: 1) There is no uniform standard for the high quality of tongue images; 2) Due to the difference of subjective feelings of practitioners, there are deviations in the subjective evaluation performance; 3) It requires huge human labor. In order to overcome the above problems, objective IQA methods based on computer image analysis have been proposed. Wang et al. (Wang and Bovik, 2006) proposed to evaluate the quality of TCM tongue images through geometric, color, and texture features. Zhang et al. (Zhang et al., 2016) proposed to extract texture features, color features, spatial, and spectral entropy features from segmented tongue images, and input them into a support vector machine-based classification model, with an accuracy of 90%. However, the artificially designed traditional image morphological features have limited description performance for image quality, and it is difficult to generalize the quality of tongue maps.

In recent years, deep learning networks have achieved significant results in image recognition by extracting deep-level features of images in a data-driven manner, demonstrating superiority over traditional hand-designed features. Deep learning technology has been widely used in the study of tongue images in various scenarios, such as tongue image segmentation (Lin et al., 2018; Xue et al., 2018), tongue diagnosis (Li et al., 2021), tongue color feature extraction (Yang and Zhang, 2018; Guangyu et al., 2021), and tongue shape recognition (Huang et al., 2010). Recently, Jiang et al. (Jiang et al., 2021) proposed a deep convolutional neural network for tongue IQA, showing that the deep features of tongue images have a better evaluation performance for tongue image quality. However, their study used the whole tongue image as the evaluation object, including the tongue body and the surrounding background area, while the information obtained by tongue diagnosis mainly comes from the tongue body (e.g., body color, body shape, tongue coating, etc.) (Giovanni, 1995), and the image information around the tongue body will have an impact on tongue quality assessment. Therefore, tongue segmentation prior to tongue IQA is a prerequisite.

Xu et al. (Xu et al., 2020) proposed a multi-task learning model to simultaneously perform tongue segmentation and tongue coating classification. An excellent segmentation may contribute to better classification, as it maximizes useful feature information corresponding to tongue regions while minimizing redundant features corresponding to nonlinguistic region information (Xu et al., 2020). However, specific classification results, especially unqualified tongue images, can provide information on features, such as color and texture, to help identify specific regions for better segmentation results. This motivates us to consider using a multi-task learning (MTL) network for simultaneous tongue segmentation and tongue IQA tasks to improve the performance of tongue IQA.

We propose a multi-task deep learning model to evaluate the quality of tongue images. First, tongue images were manually annotated as high-quality and substandard tongue datasets by Chinese physicians. Second, by augmenting the tongue segmentation subtask, we designed an MTL network for tongue IQA. Finally, we adaptively designed different task network weight coefficients between the two tasks to obtain a better tongue IQA performance. Clinical tongue images were used to demonstrate the effectiveness of our method. To our knowledge, this is the first study to use a multi-task learning framework to evaluate tongue image quality.

## 2 Materials and methods

### 2.1 Tongue image acquisition

This study was approved by the local ethics committee and the patients provided informed consent. Professional tongue image collection equipment was used to collect tongue image data from the healthy volunteers. All the collected tongue images were independently assessed as high-quality and unqualified by three professional practitioners of traditional Chinese medicine. Tongue images with inconsistent evaluation results were marked separately again, and the three TCM physicians reached a consensus on the quality evaluation results. The image quality data evaluated by multiple professional physicians will serve as the gold standard for subsequent deep network training and performance measurements.

### 2.2 Standard of image quality

**FIGURE 1:**
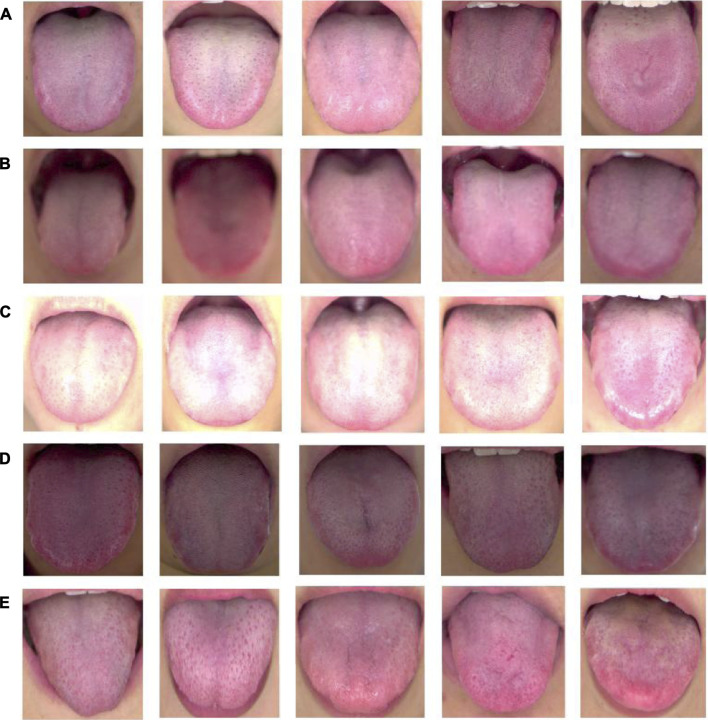
High-quality and unqualified tongue images. **(A)** high quality tongue images; **(B)**-**(E)** unqualified images; **(B)** blurred tongue images, **(C)** too brightly lit tongue images; **(D)** too dimly lit tongue images, **(E)** tongue with insufficient tongue extension.

According to the diagnosis theory of traditional Chinese medicine, the evaluation criteria of high-quality tongue images to meet the clinical needs of traditional Chinese medicine practitioners have the following characteristics (Giovanni, 1995): 1) the tongue image is clear and there is no image blurred area; 2) the light taken is naturally soft, and there is no image color distortion caused by too much brightness or darkness. 3) The tongue body was fully extended and naturally extended to the outside of the lower lip, and the surface was flat. A representative sample of high-quality tongue images is shown in Figure 1A.

In addition, we used professional tongue image acquisition equipment to obtain tongue images of the participants under different lighting conditions, exposures, and tongue protrusion conditions, as control unqualified tongue images. There were four main types of unqualified tongue images, including blurred tongue images, tongue images with too much light or insufficient light, underexposed tongue images, and tongue images with incorrect stretching postures, as shown in Figure 1B–E. Among them, shaking or vibration of the tongue during the shooting process easily leads to blurred focus, which may form a blurred picture, as shown in Figure 1B. In addition, excessive ambient light hitting the tongue surface will make the main area of the tongue too bright, and the image color will be too white, as shown in Figure 1C. As shown in Figure 1D, dark ambient light and insufficient exposure can also lead to darkening of the tongue surface, which affects clinical judgment. Tight tongue muscles and insufficient tongue extension caused by excessive tension or incorrect tongue extension posture during shooting are shown in Figure 1E.

### 2.3 Image preprocessing

**FIGURE 2:**
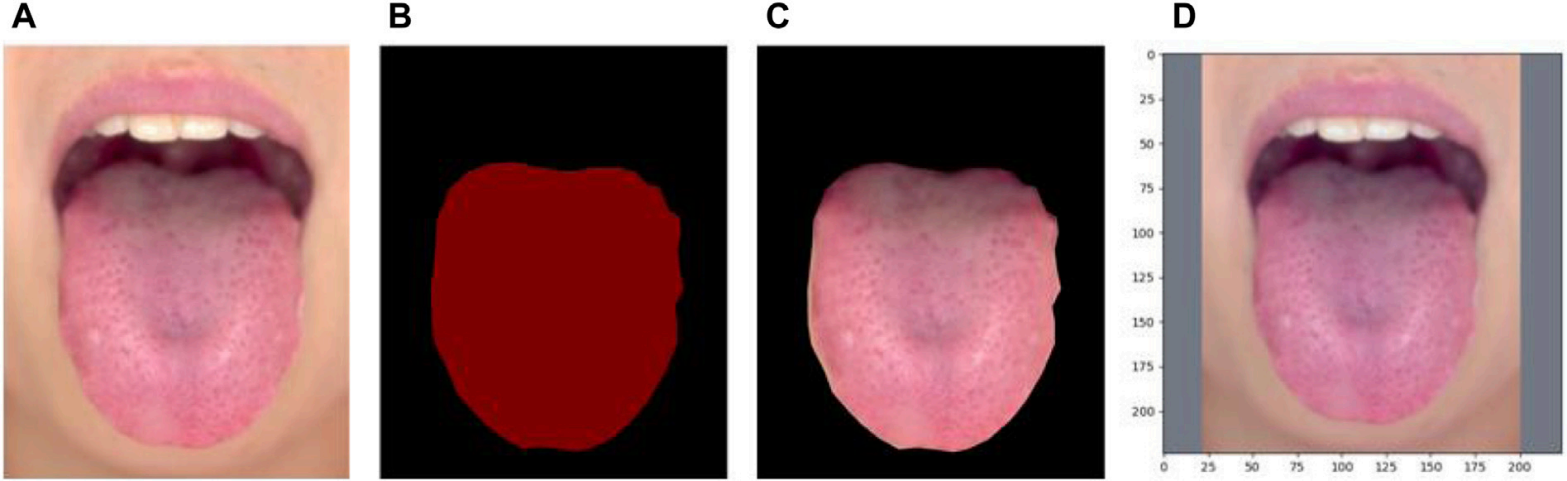
**(A)** Original image **(B)** segmentation mask **(C)** extracted tongue image **(D)** resized image.

To construct the auxiliary task of tongue segmentation in the multi-task learning network, we preprocessed the collected tongue images. First, we manually outlined the tongue region from the captured images using the Labelme software (http://labelme.csail.mit.edu/Release3.0/), as shown in Figures 2A,B. We then cut out the pixels corresponding to these contour regions in the original image, thereby extracting the tongue region image without the background, as shown in Figure 2C. Finally, we normalized the extracted tongue and face images, as shown in Figure 2D, and uniformly resized the tongue image to 224 × 224, while using random translation and rotation for data augmentation. The segmentation mask of the tongue map and the high- and low-quality labels of the tongue image previously determined by experienced Chinese physicians were used to train the deep learning network for multi-task learning.

### 2.4 The proposed framework

**FIGURE 3:**
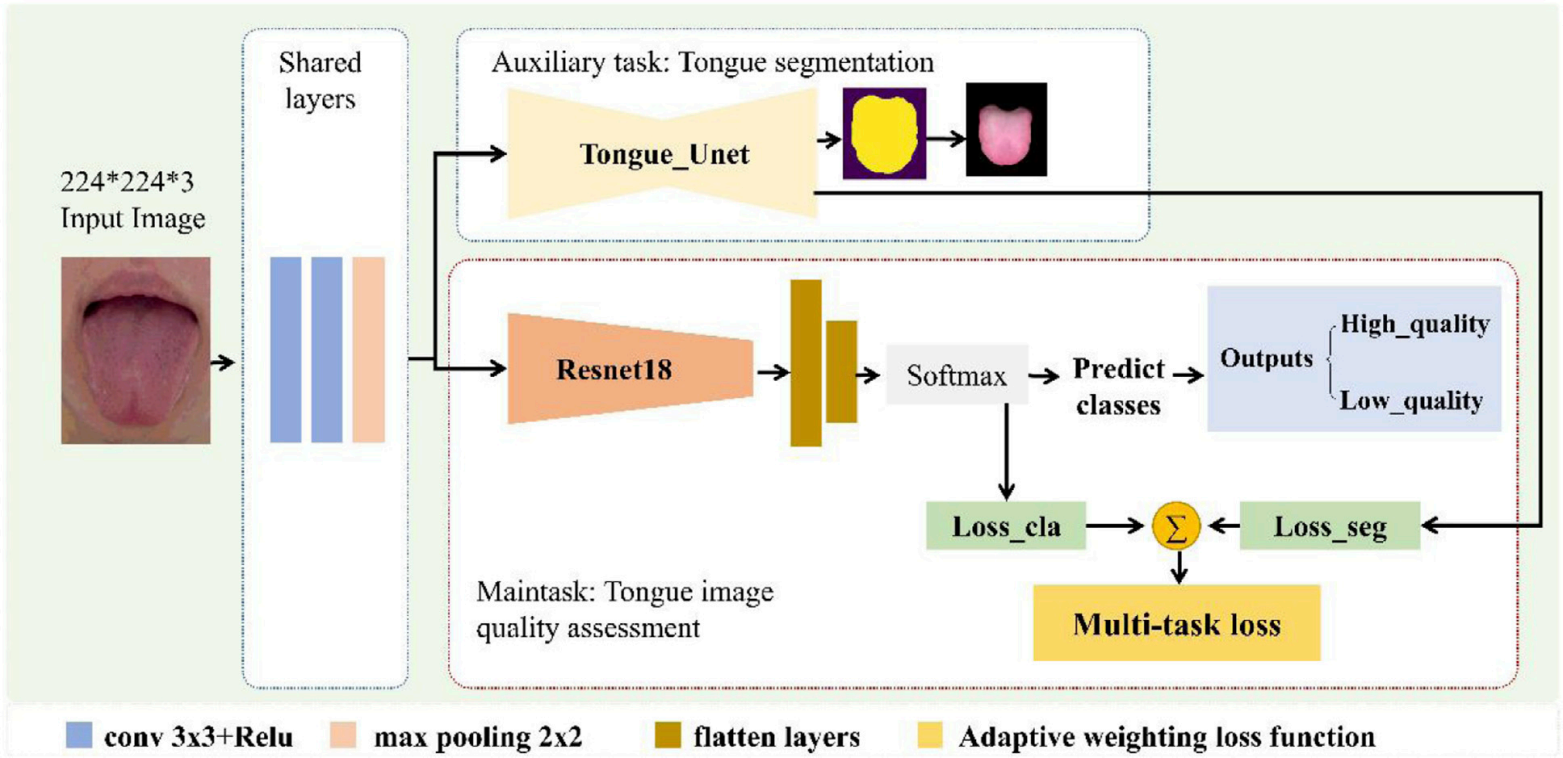
Proposed multi-task deep learning framework.

The proposed multi-task deep-learning framework is shown in Figure 3. The network architecture consists of two parts: a shared layer and a task-specific layer. Owing to the strong performance of U-net in tongue image analysis (Ruan et al., 2021), this study adopts it as the typical convolutional neural network (CNN) backbone. The purpose of the shared layer is to extract the common features between two related tasks. While the number of network parameters can be reduced, the common features can be extracted to obtain reliable representation features between tasks. The task-specific layer extracts deep features related to the respective tasks and improves the feature representation performance of the respective tasks. In addition, to balance the difference in the contribution of the two tasks to the network optimization, we designed an adaptive weighting between tasks to obtain the optimal task weight coefficient to further improve the performance of multi-task learning. Each module is described in detail in the following subsections.

#### 2.4.1 Tongue image segmentation subnetwork

**FIGURE 4:**
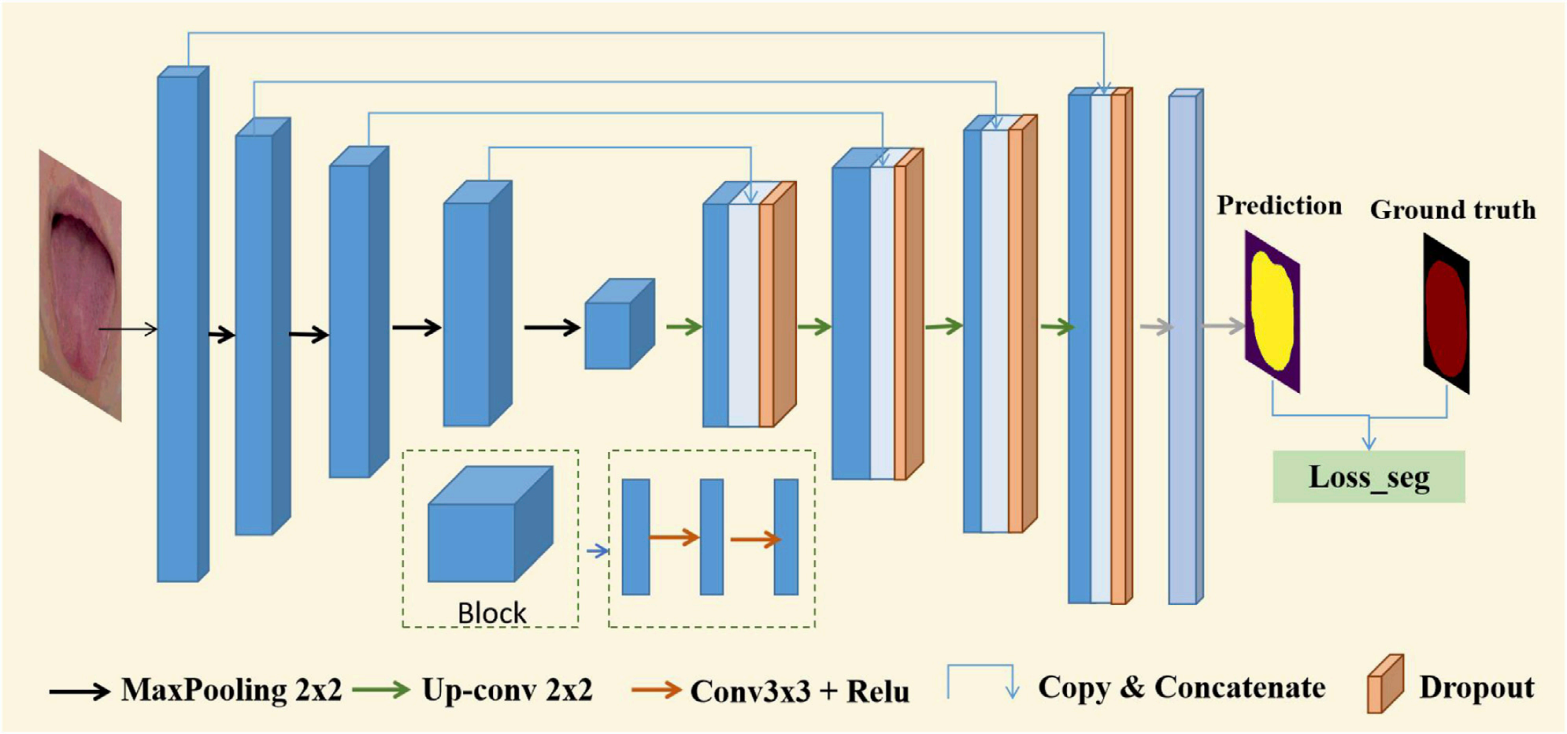
Structure of U-Net based on VGG16.

We adopted a typical U-Net (Ronneberger et al., 2015) as the baseline model, which is an image-to-image classifier based on a fully convolutional network for pixel-level prediction, as shown in Figure 4. To adapt to the segmentation of tongue images, we made the following improvements to the U-Net network structure: First, a dropout layer with a parameter of 0.5 was added. The decoder consisted of upsampling and concatenation, followed by regular convolution operations. In the symmetrical network architecture of U-Net, the encoder is on the left and the decoder is on the right. The block layers used three 3 × 3 filters and rectified linear activation functions, followed by a max-pooling layer, which reduced the dimensionality of the features and avoided overfitting. Finally, it is output through the convolutional layer and softmax function. In this tongue dataset, the tongue occupies a large part of the image, as shown in Figure 2. We used the binary cross-entropy $L_{CE}$ and dice coefficient $L_{Dice}$ as the tongue segmentation loss function $L_{seg}$ followed in (Yeung et al., 2022), which is described as follows:

$$L_{CE}=\;-\sum_{1}^{N}y_{i}\text{log}\left({\hat{y}}_{i}\right)$$ (1)

$$L_{Dice}=1-\;\frac{2*\sum_{1}^{N}\left(y_{i}*{\hat{y}}_{i}\right)}{\sum_{1}^{N}{\hat{y}}_{i}^{2}+\sum_{1}^{N}y_{i}^{2}}$$ (2)

$$L_{Seg}=\;L_{CE}+\;L_{Dice}$$ (3)

where the sums run over the N pixels, of the predicted binary segmentation pixels ${\hat{y}}_{i\;}\epsilon \;\hat{Y}$ and ground truth binary pixels $y_{i\;}\epsilon \;Y$ .

#### 2.4.2 Tongue IQA main work

In the main task of tongue image quality assessment, the encoder consists of an underlying shared layer and task-specific layer for tongue image quality classification. Shared layers were used to extract common features across tasks, and task-specific layers were used to extract deep features for tongue image quality classification, thereby mapping labels to high- and low-quality images. The specific process is to input the normalized tongue image into the network (batch_size, 3, 224, 224), then pass through the shared layer and the specific task layers based on the Resnet18 (He et al., 2016) backbone, and finally enter the fully connected layer and classifier (batch_size, 2), which are mapped to the corresponding high- and low-quality image classification labels. For the tongue image quality assessment classification task, we used cross-entropy as the loss function, as shown in Eq. 4.

$$L_{Cla}=\;-\left[y_{true}\text{log}\left(y_{pred}\right)\;+\;\left(1-y_{true}\right)\text{log}\left(1-y_{pred}\right)\right]$$ (4)

Here, $y_{pred}$ and $y_{true}$ denote the flattened predicted probabilities and ground truths of the high-quality tongue image, respectively. $1-y_{true}$ and $1-y_{pred}$ indicate the flattened predicted probabilities and ground truths of the low-quality tongue image, respectively.

#### 2.4.3 Adaptive loss function of multi-task learning

There are differences in the weights of different tasks during the optimization process in multi-task learning (Cipolla et al., 2018). Therefore, we designed an adaptive task weight coefficient to further improve the performance of multi-task learning.

Inspired by the work of Cipolla et al. (Cipolla et al., 2018) in the field of computer vision, the loss function of two tasks with the same weight in multi-task learning is shown in Eq. 5, whereas the multi-task loss function based on adaptive weighting is shown in Eq. 6.

$$L_{total}=\;L_{Cla}+\;L_{Seg}=\;L_{1}+\;L_{2}$$ (5)

$$L_{total}\;=\sum_{1}^{i=2}\left(\frac{1}{2\sigma_{i}^{2}}L_{i}+\text{log}\left(1+\sigma_{i}^{2}\right)\right)$$ (6)

To avoid negative numbers in log ( $\sigma_{i}^{2}$ ), we set the initial value to log (1+ $\sigma_{i}^{2}$ ) greater than or equal to 1. Here, $\sigma_{i}$ is the trainable hyperparameters of the $i^{th}$ task.

### 2.5 Implementation and training strategy

Our proposed model was implemented using Pytorch (Pytorch. org) and used the Adam algorithm to minimize the objective function. We used an NVIDIA TITAN RTX graphics card with 24GB memory. The initial learning rate is set to 1e-4, weight decay is set to 5e-4, and batch size were set to 4. The performance specifications of the computer are as follows: CPU, Intel(R) Xeon(R) Gold 5,118. RAM is 64.0 GB. The GPU was an NVIDIA TITAN RTX GPU. The basic implementation code for this study is available at GitHub: *https://github.com/yanyan121/MTL_Tongue_IQA* .

In addition, we adopted U-Net as the backbone network for multi-task learning because of its excellent performance in image segmentation (Yeung et al., 2022). The weights of the model were obtained from pretraining on ImageNet (Russakovsky et al., 2015), which has a large dataset, rich categories, and great versatility. Therefore, the weight trained by ImageNet was used as the initial value of our model to further train the classification task for tongue image quality assessment. Specifically, at the beginning of training, we chose to freeze the encoder network weights, train the decoder network weights and classification network weights for 10 epochs, and train the last 40 epochs with the unfrozen weights using the loaded training learning rate.

## 3 Experimental results

### 3.1 Experiment setup and evaluation metric

The number of images used for tongue image quality assessment was 1,014, and the number of images in each class was high quality (546 images) and poor quality (468 images). The tongue images were marked in advance by professionals, and the marked tongue images were subsequently used as the training (70%), validation (15%) sets and testing (15%) sets. For tongue segmentation subtask, we used Dice similarity coefficient (DSC), Jaccard index (JI) (Bertels et al., 2019), Mean intersection over union (MIoU), frequency weighted intersection over union (FWIoU) for quantitative evaluation. These metrics were calculated as follows:

$$DSC\left(pred,true\right)=\;\frac{2*TP}{2*TP+FP+FN}$$ (7)

$$JI\left(pred,true\right)=\;\frac{TP}{TP+FN+FP\;}$$ (8)

$$MIoU\left(pred,true\right)=\;\frac{1}{2}*\left(\frac{TP}{TP+FN+FP\;}+\frac{TN}{TN+FN+FP\;}\right)$$ (9)

$$FWIoU\left(pred,true\right)=\;\frac{1}{TP+FP+TN+FN}\left[\frac{TP*\left(TP+FN\right)}{TP+FP+FN}+\frac{TN*\left(TN+FP\right)}{TN+FN+FP\;}\right]$$ (10)

DSC is used to measure the similarity of two sets, whereas JI compares members for two sets to see which members are shared and which are distinct. Also known as the JI, IoU is a statistic used for comparing the similarity and diversity of sample sets. In semantics segmentation, it is the ratio of the intersection of the pixel-wise classification results with the ground truth, to their union. MIoU is the class-averaged IoU. FWIoU is a frequency-weighted IoU. For tongue quality classification, we employed accuracy, precision, recall, and F1-score for quantitative evaluation.

$$Accuracy\left(pred,true\right)=\frac{TP+TN}{TP+TN+FP+FN}$$ (11)

$$Precision\left(pred,true\right)=\frac{TP}{TP+FP}$$ (12)

$$Recall\left(pred,true\right)=\frac{TP}{TP+FN}$$ (13)

$$F1\;Score\left(pred,true\right)=\frac{2}{1/Precision+1/Recall}$$ (14)

where TP, FP, TN, and FN represent true positives, false positives, true negatives, and false negatives, respectively. In the classification task, it represents the prediction and ground truth, whereas in the segmentation task, it represents the pixel-wise labels.

To evaluate the effectiveness of the proposed method, several ablation experiments were conducted. The differences between ablation models are listed in Table 1. STL_original_images, MTL_equal_weight, and MTL_adaptive_weight used original tongue images as shown in Figure 2A. To assess the interference of the surrounding background, we compared the original tongue images and the tongue region without the background on the performance of tongue image quality classification.

**TABLE 1:** Ablation models. STL: single task learning; MTL: multi-task learning; Cla: classification; Seg: segmentation.

Models	Task	Input images
STL_OTI	Cla	Original tongue images (Figure 2A) (OTI)
STL_ETI	Cla	Extracted tongue image (Figure 2B) (ETI)
MTL_equal_weight	Cla + Seg	Original images (Figure 2A)
MTL_adaptive_weight	Cla + Seg	Original images (Figure 2A)

### 3.2 Performance comparison of different methods

We compare the performance of our method with state-of-the-art deep learning tongue image quality assessment and tongue image segmentation research. Jiang et al. (Jiang et al., 2021) is a recently proposed tongue image quality assessment method based on deep learning network, which is a binary classification task performed by the ResNet architecture. Due to the discrepancy between datasets, the accuracy of the method tested in our dataset is 0.813, while the accuracy of our proposed multi-task learning based tongue image quality assessment is 0.890, an improvement of 0.077. Furthermore, in the auxiliary task of tongue image segmentation, we compare our method with two state-of-the-art segmentation methods with network architectures Deeptongue (Lin et al., 2018) and DeepLabV3 (Xue et al., 2018), respectively. As shown in Table 2, under the multi-task learning framework, our proposed tongue image segmentation method has a certain degree of improvement compared with the current tongue segmentation methods Deeptongue and DeepLabV3. The main reason for the performance improvement should be the mutual promotion of associated tasks in multi-task learning, thereby promoting the improvement of single-task performance.

**TABLE 2:** Performance comparison of different methods.

	Classification (mean ± sd)				Segmentation(mean ± sd)			
Models	Accuracy	Precision	Recall	F1-score	DSC	JI	MIoU	FWIoU
ResNet_base (Jiang et al., 2021)	0.813 ± 0.041	0.811 ± 0.027	0.801 ± 0.048	0.807 ± 0.031	--	--	--	--
Deeptongue (Lin et al., 2018)	--	--	--	--	0.9647 ± 0.0402	0.9581 ± 0.2373	0.9569 ± 0.0715	0.9573 ± 0.1964
Deeplabv3 (Xue et al., 2018)	--	--	--	--	0.9651 ± 0.0136	0.9617 ± 0.4013	0.9577 ± 0.0116	0.9579 ± 0.2399
MTL_adaptive weight(ours)	0.890 ± 0.018	0.873 ± 0.034	0.899 ± 0.035	0.870 ± 0.017	0.9673 ± 0.0015	0.9711 ± 0.0044	0.9681 ± 0.0604	0.9693 ± 0.0170

### 3.3 Performance of ablation study in the proposed method

Table 3 shows the performance comparison of single-task learning (STL) and multi-task learning based on tongue image segmentation, and the performance comparison of using two different loss-weighing strategies in tongue image quality assessment. We found that multi-task learning with the same weight policy yielded better performance than single-task learning with extracted tongue images. Furthermore, in the framework of multi-task learning, the adaptive weighting strategy demonstrates better performance than the equal weighing strategy. Compared to single-task learning using original tongue images, the proposed framework achieved a significant improvement of 0.074 in accuracy.

**TABLE 3:** Performance of ablation study in the proposed method.

	Classification (mean ± sd)				Segmentation(mean ± sd)			
Models	Accuracy	Precision	Recall	F1-score	DSC	JI	MIoU	FWIoU
STL_OTI (Xu et al., 2020)	0.816 ± 0.035	0.819 ± 0.015	0.803 ± 0.019	0.810 ± 0.023	0.9657 ± 0.0008	0.9691 ± 0.3395	0.9672 ± 0. 2613	0.9677 ± 0.0686
STL_ETI	0.878 ± 0.027	0.864 ± 0.031	0.822 ± 0.025	0.842 ± 0.014	--	--	--	--
MTL_equal weight	0.879 ± 0.021	0.833 ± 0.028	0.885 ± 0.037	0.858 ± 0.026	0.9662 ± 0.0020	0.9698 ± 0.3347	0.9675 ± 0.1300	0.9678 ± 0.2701
MTL_adaptive weight (ours)	0.890 ± 0.018	0.873 ± 0.034	0.899 ± 0.035	0.870 ± 0.017	0.9673 ± 0.0015	0.9711 ± 0.0044	0.9679 ± 0.0604	0.9707 ± 0. 1278

**FIGURE 5:**
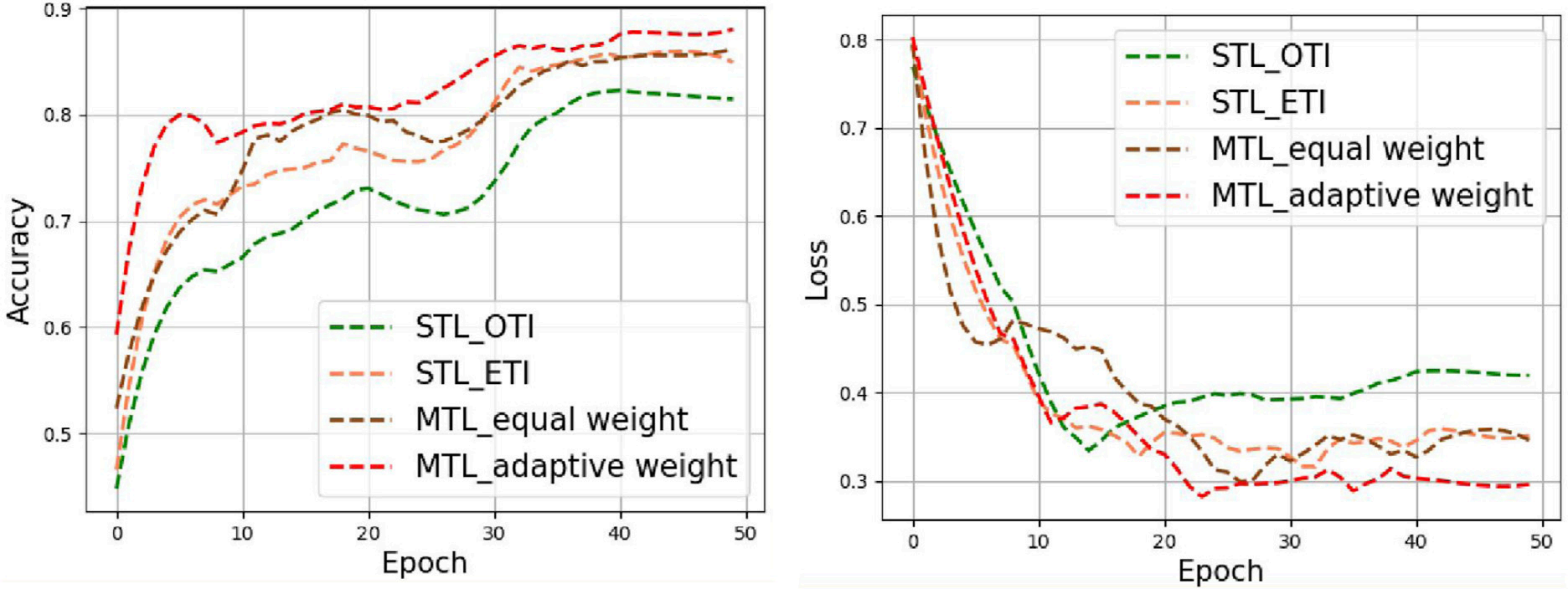
Accuracy and loss curves for the different methods in tongue images.

Figure 5 shows the accuracy and loss curves of several typical tongue image-quality assessment models. Throughout the testing process, our proposed multi-task learning framework (MTL_adaptive weight) consistently showed better performance than single-task learning of original tongue images, extracted tongue images, and multi-task learning based on an equal-weight strategy.

**FIGURE 6:**
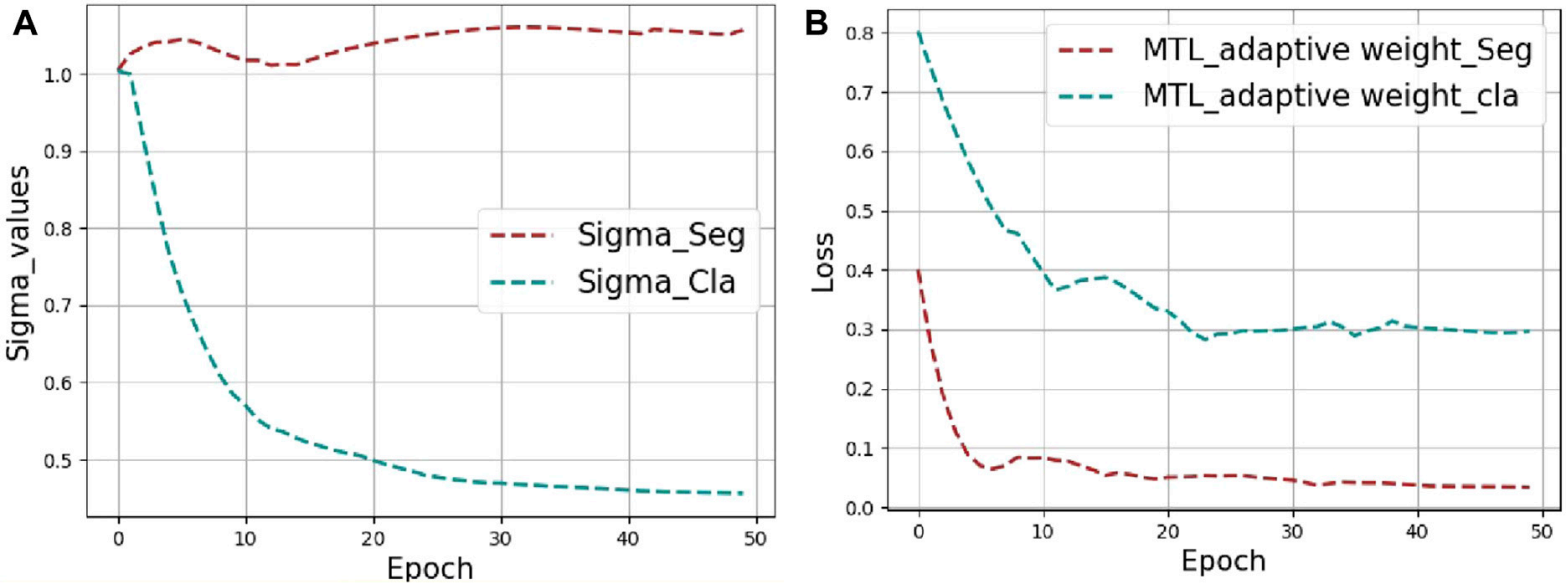
The visualization results of MTL_adaptive weight strategy. **(A)** Visualization of the weight values (Sigma_Cla = *σ1*, Sigma_Seg = *σ2*) changing during training. **(B)** Loss curves for segmentation and classification tasks.

These two hyperparameters were set before training and the initial value is set to 1. We have added a graph showing how σ1 and σ2 change with epoch during training, as shown in Figure 6A. After the curve converges and stabilizes, the weights of the classification and the segmentation are finally 0.45 and 1.05, respectively. For heterogeneous MTL problems (e.g., the segmentation and classification of tongue images) that contain tasks of different types, following (Maninis et al., 2019; Vandenhende et al., 2022) different measurement methods lead to a large difference in the calculated scalar, as shown in Figure 6B, where the loss value of the classification task is much larger than that of the segmentation task. These two tasks loss values are regularized by adaptive weighting, and then the back propagation gradient of the segmentation task is increased. In this way the auxiliary ability of the segmentation task is improved.

### 3.4 Visualization

**FIGURE 7:**
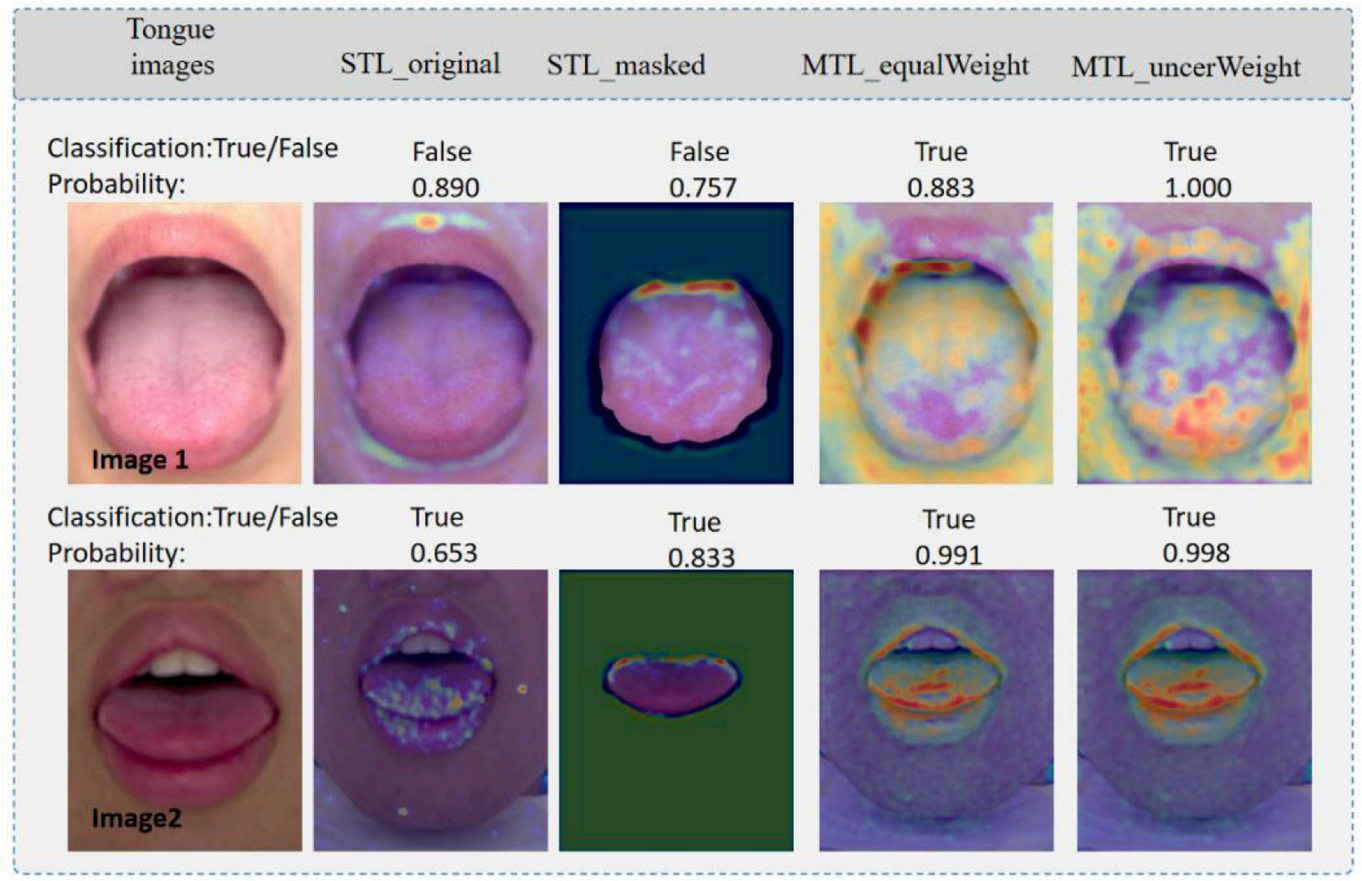
Visualization of saliency maps. Image1: high quality tongue image; and Image2. True indicates that the prediction is correct, and False indicates that the prediction is wrong.

Figure 7 shows a heatmap visualization using the gradient-weighted class activation map (Grad-CAM) (Selvaraju et al., 2017), which reflects the main features of the regions that contribute to the prediction results. Darker red areas and brighter pixels indicate areas in which different models are focused. Precisely, the first line represents high-quality tongue images, whereas the second line represents low-quality tongue images. “True” indicates that the prediction is correct and “False” indicates that the prediction is incorrect. Moreover, the numbers (e.g., 0.890) indicate the probability value of the predicted outcome of the tongue image quality classification in Figure 7. The single-task model pays more attention to the tongue, whereas the multi-task model pays more attention to the main part of the tongue image and its boundaries. By comparison, it can be found that the feature extractor in the multi-task model can better capture the information of the main part of the tongue body and tongue boundary area. The proposed multi-task model can focus on more comprehensive feature regions, which improves the quality assessment performance of the multi-task model.

**FIGURE 8:**
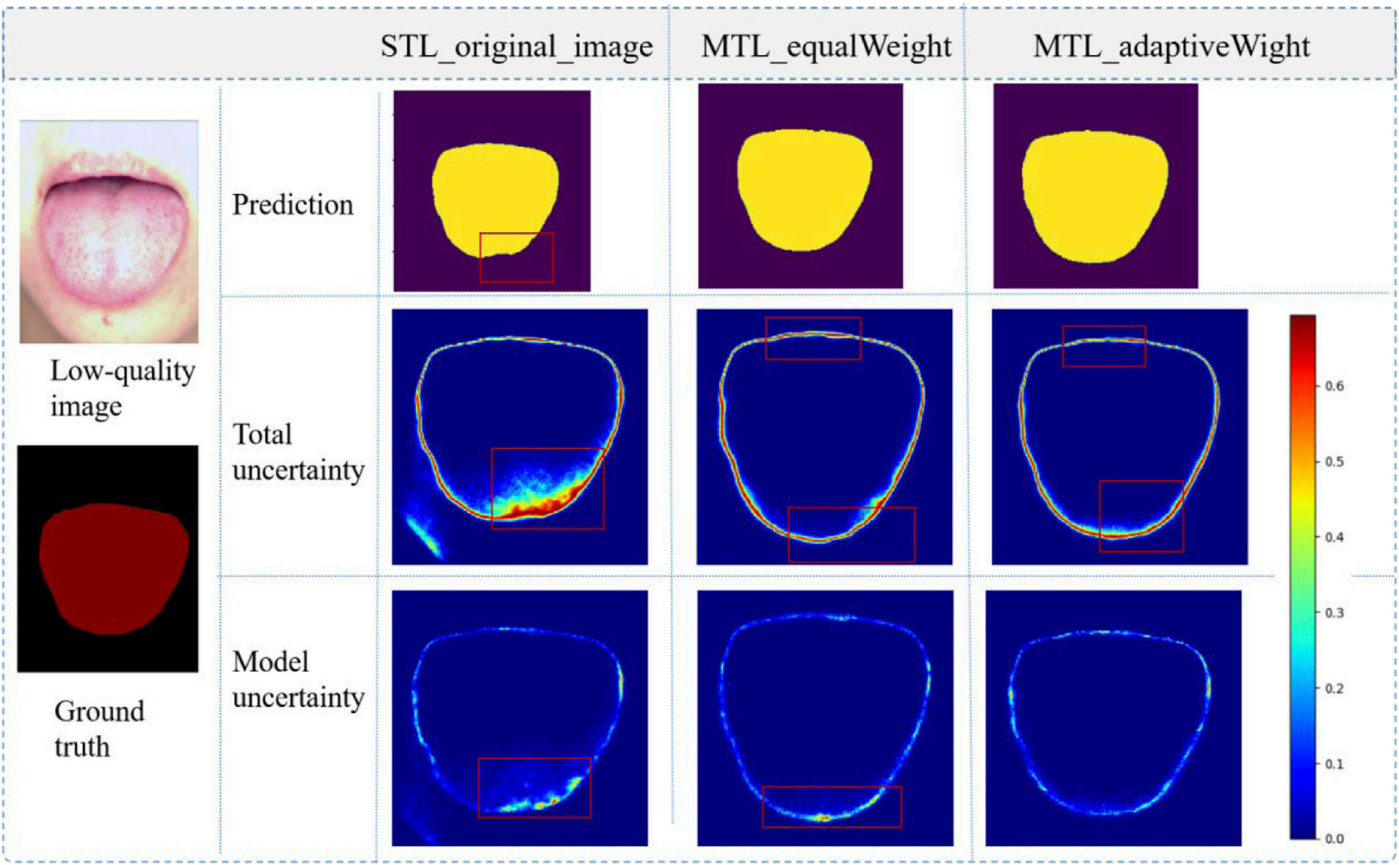
Visualization of tongue segmentation, prediction: prediction results; Total uncertainty: data uncertainty and model uncertainty; The value range of uncertainty maps is between 0 and 1, and a larger value represents a higher degree of uncertainty.

In the segmentation task, by quantifying the performance of different models by DSC and JI, it was found that the segmentation performance was also slightly improved. Bayesian neural networks with Monte Carlo dropout (MC dropout) can obtain uncertainty estimations (Gal and Ghahramani, 2015), which is useful and powerful. Using Dropout = 0.5 at test time, we can visualize the uncertainty of the segmentation boundaries. Figure 8 shows the visualization of the uncertainty in the segmentation results. The redder the color, the higher the uncertainty value of the output of the region. The overall certainty is found to be higher in the adaptive multi-task learning model; thus, the results are more reliable. More importantly, we found that the evaluation of image quality is closely related to the tongue boundary, and the higher the certainty of the segmented boundary, the higher is the accuracy of quality classification.

## 4 Discussion

In this study, our proposed multi-task learning model mainly addresses the clinical problem in tongue image quality assessment. The performance of tongue image quality assessment was further improved by adding tongue body segmentation as an auxiliary task. To the best of our knowledge, this is the first study to use multi-task learning for tongue image quality assessment. Compared with the existing deep learning tongue image quality assessment research (Xu et al., 2020), our multi-task learning method achieved better results in tongue image quality assessment. The performance was greatly improved, and the auxiliary task of our multi-task learning could output the segmented tongue, which further facilitates subsequent tongue diagnosis. Therefore, this method provides a good reference for the application of artificial intelligence in tongue diagnosis.

Multi-task learning has been widely used in the field of artificial intelligence, especially in the segmentation and classification of medical images (Zhang et al., 2021). We applied multi-task learning to tongue image quality assessment, mainly considering that tongue map quality and tongue body segmentation are two related tasks. According to the multi-task learning theory, for two related tasks, multi-task learning can further boost the performance of both tasks (Ranjan et al., 2018; Xu et al., 2020). In addition, our multi-task learning further considers the different weights of different tasks in the optimization process. By optimizing the design of adaptive weight coefficients, the performance of tongue map quality evaluation and tongue segmentation was further improved.

It should be noted that there were certain differences between the tongue quality assessment in this study and the general image quality assessment (Zhu et al., 2020; Ma and Fang, 2021). For a general image quality assessment, more information, such as image color distortion and blurring, should be considered, which has the same requirements as our tongue map quality assessment. However, for the special tongue diagnosis, incomplete tongue extension and an excessive or too dark environment will lead to a low-quality tongue map; thus, making our evaluation of tongue map quality incapable of completely copying the general quality evaluation method. For this reason, we used the tongue images collected by the joint identification of three TCM physicians to construct high-quality and low-quality tongue images as the learning samples of the multi-task learning deep network.

This study had certain limitations. First, low-quality images in this study were obtained by virtually changing the shooting scene, not from clinical practice, and may not completely simulate all low-quality image situations. During follow-up, tongue image data may be obtained from clinical practice and the performance of this method may be independently verified. Second, our multi-task learning required the construction of auxiliary segmentation subtasks, which demands the manual delineation of practitioners and as a result brings a great workload on the clinic. In the future, we will consider integrating unsupervised or self-supervised segmentation tasks into a multi-task learning deep network to reduce the clinical workload in the preprocessing stage. In addition, the module we designed was only a postprocessing image evaluation after image acquisition. Obtaining the results of image quality evaluation in real time during the image acquisition stage can greatly improve the success rate of high-quality tongue image acquisition. Therefore, the focus of future studies is to integrate this method into real-time tongue image acquisition equipment for the real-time identification of tongue image quality.

## 5 Conclusion

In this study, we propose a multi-task deep learning model for tongue image quality assessment. By adding the tongue segmentation subtask, the experimental results showed that the performance of the multi-task learning network for tongue image quality assessment was significantly improved. In addition, multi-task learning deep network could output tongue segmentation regions, which could facilitate subsequent clinical tongue diagnosis. We believe that the research method in this study has great value as a reference for the clinical application of tongue diagnosis.

## Data Availability

The original contributions presented in the study are included in the article/Supplementary Material, further inquiries can be directed to the corresponding authors.
